# Maturation of the Acute Hepatic TLR4/NF-κB Mediated Innate Immune Response Is p65 Dependent in Mice

**DOI:** 10.3389/fimmu.2020.01892

**Published:** 2020-08-21

**Authors:** Miguel A. Zarate, Leanna M. Nguyen, Robyn K. De Dios, Lijun Zheng, Clyde J. Wright

**Affiliations:** Section of Neonatology, Department of Pediatrics, Children's Hospital Colorado, University of Colorado School of Medicine, Aurora, CO, United States

**Keywords:** liver, innate immunity, LPS, NF-κB, *Rela* (p65), *Nfkb1* (p50)

## Abstract

Compared to adults, neonates are at increased risk of infection. There is a growing recognition that dynamic qualitative and quantitative differences in immunity over development contribute to these observations. The liver plays a key role as an immunologic organ, but whether its contribution to the acute innate immune response changes over lifetime is unknown. We hypothesized that the liver would activate a developmentally-regulated acute innate immune response to intraperitoneal lipopolysaccharide (LPS). We first assessed the hepatic expression and activity of the NF-κB, a key regulator of the innate immune response, at different developmental ages (p0, p3, p7, p35, and adult). Ontogeny of the NF-κB subunits (p65/p50) revealed a reduction in *Rela* (p65) and *Nfkb1* (p105, precursor to p50) gene expression (p0) and p65 subunit protein levels (p0 and p3) vs. older ages. The acute hepatic innate immune response to LPS was associated by the degradation of the NF-κB inhibitory proteins (IκBα and IκBβ), and nuclear translocation of the NF-κB subunit p50 in all ages, whereas nuclear translocation of the NF-κB subunit p65 was only observed in the p35 and adult mouse. Consistent with these findings, we detected NF-κB subunit p65 nuclear staining exclusively in the LPS-exposed adult liver compared with p7 mouse. We next interrogated the LPS-induced hepatic expression of pro-inflammatory genes (*Tnf*, *Icam1, Ccl3*, and *Traf1*), and observed a gradually increase in gene expression starting from p0. Confirming our results, hepatic NF-κB subunit p65 nuclear translocation was associated with up-regulation of the *Icam1* gene in the adult, and was not detected in the p7 mouse. Thus, an inflammatory challenge induces an NF-κB-mediated hepatic innate immune response activation across all developmental ages, but nuclear translocation of the NF-κB subunit p65 and associated induction of pro-inflammatory genes occurred only after the first month of life. Our results demonstrate that the LPS-induced hepatic innate immune response is developmentally regulated by the NF-κB subunit p65 in the mouse.

## Introduction

Worldwide, neonates suffer a disproportionate burden of infection, sepsis and related morbidities and mortality when compared with older children and adults (1, 2). This is consistent with pre-clinical reports indicating that neonatal animals are highly sensitive to endotoxic shock, often demonstrating mortality at exposures 10 times lower than similarly exposed adults (3–11). This finding has been attributed to an immature immune system that is biased against a pro-inflammatory innate immune response during the fetal and perinatal period (12–17). It is thought that at this life stage, the innate immune system has a primary role of defense against diverse range of antigens since there is a limited exposure to antigens that can activate adaptive mechanisms (18). Neonates need a continuous microbial antigen exposure to gradually mature to a Th1-type, pro-inflammatory innate immunity during infancy (19). This antigen education is necessary to obtain a fully functional and mature innate immune system (20), but when this process starts or how it develops have not been fully elucidated.

The NF-κB transcription factor plays a relevant role in regulating the innate immune response. NF-κB activation is induced by degradation of the inhibitory proteins IκBα and IκBβ, and nuclear translocation of its subunits p65, p50, cRel, RelB, and p52 (21–23). Particularly, the NF-κB p65 subunit contains a C-terminal transcriptional activation domain that in necessary for its transcriptional activity (24). This domain undergoes post-translational modification, which affects NF-κB transcriptional activity through its association with interacting proteins leading to the activation of pro-inflammatory genes (25). Of note, in neonatal hepatic lysates, NF-κB p50 subunit is more abundant than p65 (26). This observation has potential implications for the neonatal hepatic innate immune response, as p50 has been reported to induce immunotolerance due to a lack of transcription activation domain in other experimental settings (27). As we have learned about the NF-κB transcriptomic, subunit p65 can bind to the promoter region of different pro-inflammatory genes such as *TNF, IL1*β*, MIP1*α*, ICAM*, and *TRAF1* (28–33). However, whether this subunit exerts the same effects at different developmental time points during an inflammatory challenge has not been fully elucidated.

During fetal development and perinatal life, the liver plays a key role in regulating the innate immune response due to its essential role in clearing antigens from the systemic circulation (34–37) via the portal vein, the biggest blood supply from the gut (38, 39). It has been reported that the liver uniquely contributes to the NF-κB-mediated innate immune response to an inflammatory challenge through the activation of pro-inflammatory cytokines (26, 40). Furthermore, we have observed that this response resembles a Th1-type in adults when compared with neonates hours after intraperitoneally (IP) LPS exposure (26). However, whether this difference can be linked to the actions of the p65 subunit expression and/or nuclear translocation has never been explored. Likewise, describing how the hepatic innate immune response to LPS evolves at different developmental time points remains incompletely characterized. Therefore, a better understanding of the mechanisms that regulates the acute hepatic NF-κB activation in the neonate and adult could reveal therapeutic targets against sepsis and prevent morbidity and mortality associated with early life infection. In this work, we proposed that the NF-κB-mediated pro-inflammatory acute hepatic innate immune response is developmentally regulated due to the subunit p65 actions in a murine model of endotoxemia.

## Materials and Methods

### Ethical Approval

All procedures were approved by the University of Colorado Institutional Animal Care and Use Committee (00457) and performed in compliance with the American Association for Accreditation for Laboratory Animal Care at the Perinatal Research Center at the University of Colorado School of Medicine (Aurora, CO, USA).

### Murine Model of Endotoxemia

Newborn (P0), neonatal (P3, P7), juvenile (P35), and adult (> 10 weeks) ICR male mice (Taconic Biosciences, Rensselaer, NY, USA) (*n* = 5–6/group) were exposed to a sublethal dose of intraperitoneal (IP) LPS (5 mg/kg; L2630, Sigma-Aldrich, St. Louis, MO, USA), as previously reported by our group (26, 40–42). Similar number of animals from each litter were included as a control (unexposed) group (P0, P3, P7, P35, and adult). One hour after the experiment, animals were euthanized with an IP overdose of sodium pentobarbital, and liver regions were collected as described previously (42), snap frozen in liquid nitrogen, and stored at −80°C.

### Hepatic Nuclear and Cytoplasmic Protein Extraction

Liver tissues from both LPS and control groups were homogenized using the Bullet Blender (NextAdvance, Troy, NY, USA), and protein lysates were collected and kept in T-PER buffer (ThermoFisher Scientific, Waltham, MA, USA). Hepatic cytosolic and nuclear extracts were prepared with the NE-PER kit (ThermoFisher Scientific, Waltham, MA, USA).

### mRNA Extraction and Quantitative Real Time-PCR

Hepatic mRNA was collected using the RNeasy Mini Kit (Qiagen, Valencia, CA, USA), and assessed for purity and concentration using the NanoDrop (ThermoFisher Scientific, Waltham, MA). mRNA was converted into cDNA using the Verso cDNA synthesis Kit (Thermo Scientific, Waltham, MA, USA). Relative mRNA levels were evaluated by quantitative real-time PCR using the TaqMan gene expression system (Applied Biosystems, Foster City, CA, USA). Gene expressions of *Icam1* (Mm00516023_m1)*, Il1b* (Mm01336189_m1)*, Ccl3* (Mm99999057_m1)*, Nfkb1* (Mm00476361_m1)*, Rela* (Mm00501346_m1)*, Tnf* (Mm00443258_m1), and *Traf1* (Mm00493827_m1) genes were assessed with predesigned exon-spanning primers using the StepOnePlus Real Time PCR System (Applied Biosystems, Foster City, CA, USA). We used the murine housekeeping gene *18S* to normalize RT-qPCR results and quantification was performed using the cycle threshold (ΔΔCt) method as described previously. Data are expressed as fold change relative to the mean in the control group.

### Western Blot Analysis

Whole lysates, and cytosolic and nuclear extracts were electrophoresed on a 4–12% polyacrylamide gel (Invitrogen, Carlsbad, CA, USA) and proteins were transferred to an Immobilon-P membrane (MilliporeSigma, Burlington, MA, USA). Membranes were blotted with antibodies against the NF-κB subunits p65 (1:1000, #6956 and #8242, Cell Signaling, Danvers, MA, USA), p50 (1:1000, ab32360, Abcam, Cambridge, MA, USA), p105 (1:1000, #13586, Cell Signaling, Danvers, MA, USA), and p100/p52 (1:1000, #4882, Cell Signaling, Danvers, MA, USA); and NF-κB inhibitory proteins IκBα (1:1000, #4814, Cell Signaling, Danvers, MA, USA), and IκBβ (1:1000, PA1-32136, Invitrogen, Carlsbad, CA, USA). For loading controls in our assays, we used β-actin (1:1000, #3700, Cell Signaling, Danvers, MA, USA) for cytosolic extracts; lamin B (1:1000, SC- 6217, Santa Cruz Biotechnology, Dallas, TX, USA) for nuclear extracts; and total hepatic protein staining for p105 protein expression (Supplemental Figure 1). Blots were imaged using the Li-Cor Odyssey® Fc imaging system (Li-Cor, Lincoln, NE, USA) and densitometric analysis was conducted using Image Studio version 4.0 (Li-Cor, Lincoln, NE, USA). Western blot assays were performed in 8 lanes where we were able to compare all developmental time points in single and across blots. Although some blots were not in chronological order, this was the most objective way to analyze and interpret our data correctly.

### Immunofluorescence

IP LPS-exposed and control livers from p7 and adult mice were fixed with 4% paraformaldehyde, paraffin-embedded, and stained against NF-κB p65 subunit. Briefly, after antigen retrieval (antigen unmasking solution, H-3301, Vector Laboratories, Burlingame, CA, USA), permeabilization (0.5% Triton X), and quenching with 100 mM glycine and 0.5% Pontamine Sky Blue (Chicago Sky Blue 6B, C8679-25G, Sigma-Aldrich, St. Louis, MO, USA), 5 μm sections were blocked with Sea Blocking (#37527, ThermoFisher Scientific, Waltham, MA) for 40 min, and Fc Receptor Blocking (NB309-15, Innovex Biosciences, Richmond, CA, USA) for 30 min. Sections were subsequently incubated with anti-p65 antibody (1:100, NF-κB p65 XP®, #8242, Cell Signaling, Danvers, MA, USA) at 4°C overnight. The following day, sections were incubated with secondary antibody (1:200, Alexa Fluor 488 donkey anti-rabbit, A-21206, ThermoFisher Scientific, Waltham, MA) for 1 h at room temperature. Finally, sections were mounted with DAPI (1:1000, D9542, Sigma-Aldrich, St. Louis, MO, USA) and imaged with an IX83 microscope and DP80 camera using CellSens software (Olympus Life Science, Waltham, MA, USA).

### Evaluation of Nuclear NF-κB Binding by EMSA

IRDye 700 phosphoramidite-labeled oligonucleotides with the consensus sequence for NF-κB (5′-AGTTGAGGGGACTTTCCCAGGC-3′; 3′-TCAACTCCCCTGAAAGGGTCCG-5′) (829-07924, Li-cor, Lincoln, NE, USA) was used as a probe to evaluate NF-κB binding ability. In order to identify the NF-κB subunit proteins in the binding complex (supershift expression), 3 different p50 (ab32360, Abcam, Cambridge, MA, USA; #13586, Cell Signaling, Danvers, MA, USA; and #90275, Millipore, Burlington, MA, USA) or a p65 (8242, Cell Signaling, Danvers, MA, USA), subunit antibodies were incubated with nuclear proteins for 25 min at 4°C prior to the addition of the labeled probe.

### Chromatin Immunoprecipitation (ChIP) Assay

We used the lateral left lobe of the liver for this assay. Chromatin was prepared using the EZ-Magna ChIP^TM^ G – Chromatin Immunoprecipitation tissue kit (Millipore, Burlington, MA, USA) with sonicated tissues in nuclear lysis buffer (Millipore, Burlington, MA, USA) as described previously (41). Ten μg of chromatin was diluted to a total volume of 500 μL in dilution buffer. We used antibodies against rabbit IgG (1:10, PP64, Millipore, Burlington, MA, USA), NF-κB p65 subunit (1:25, SC-372, Santa Cruz Biotechnology, Dallas, TX, USA), and RNA Polymerase II clone CTD4H8 (1 mg/mL, 05-623B, Millipore, Burlington, MA, USA) as positive control. All antibodies had an overnight incubation at 4°C. We performed RT-qPCR for *Icam1* gene enrichment with SYBR green ROX qPCR Mastermix (330523, Qiagen, Valencia, CA, USA) and pre-designed *Icam1* (F: TGGTGGGTTAAAGAGGCTTG; R: CAGGTGAGTCCGGAGAGAAG) promoter spanning primers (Integrated DNA Technologies, Coralville, IA, USA)1. Results were expressed as percent input average.

### Statistical Analysis

Statistical analysis was conducted with GraphPad Prism 8 software (GraphPad, San Diego, CA, USA). Primary cytokine gene expression and the NF-κB ontogeny data were analyzed by 1-way ANOVA (developmental time point as main factor) and differences between groups was determined by the Dunnet *post-hoc* test. IP LPS-exposed NF-κB signaling and ChIP data (LPS vs. CTR) was analyzed by Student's *T*-test. Statistical significance was declared at *P* < 0.05.

## Results

### Hepatic NF-κB p65 Subunit Gene and Protein Expression Is Developmentally Regulated

**Figure 1:**
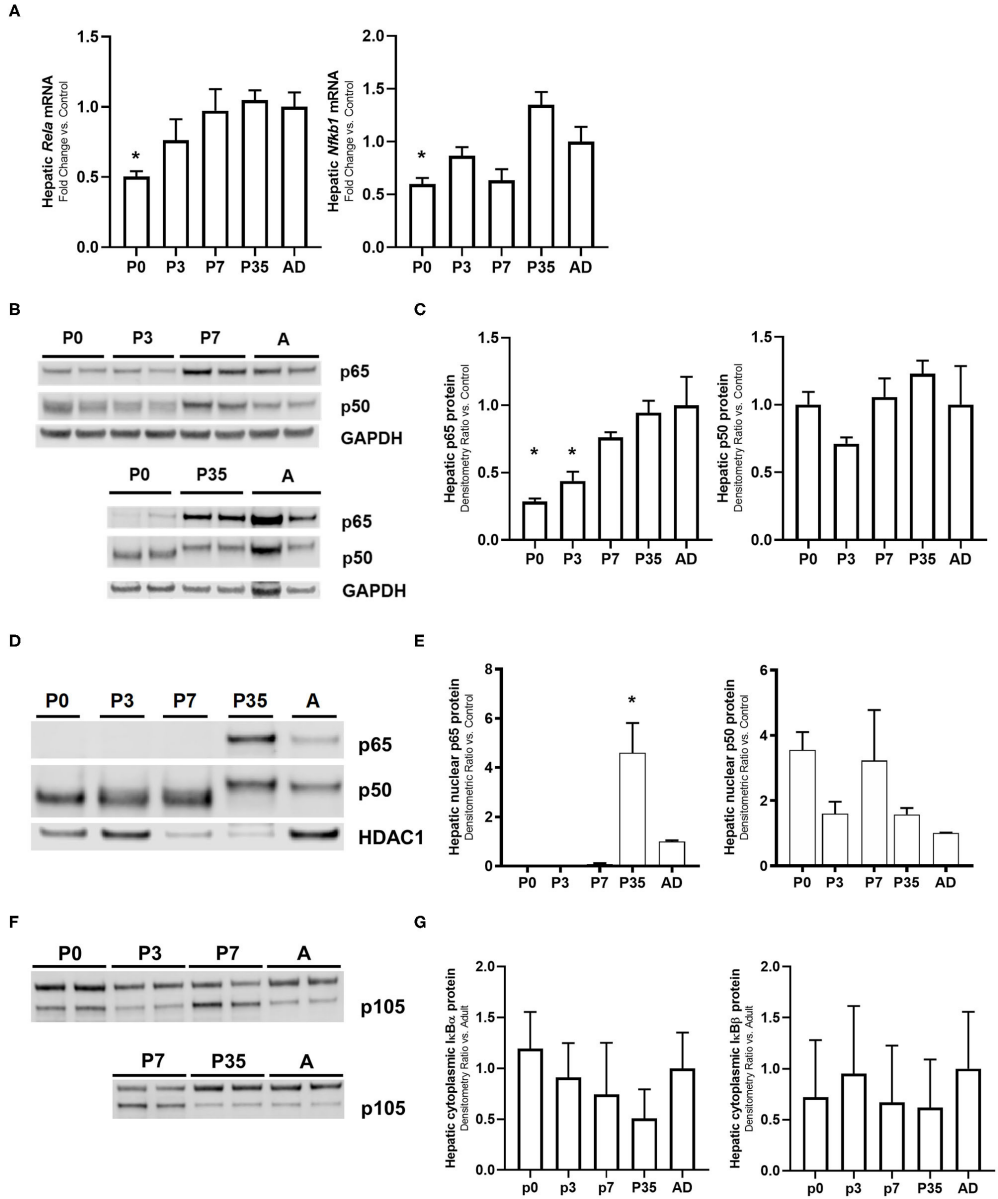
Ontogeny of the NF-κB subunits shows that p65 protein is developmentally regulated. Unexposed mouse livers from different developmental time points were collected and measured for hepatic **(A)** *cRela* and *Nfkbl* gene expression, **(B,C)** whole cell lysate and **(D,E)** nuclear protein p65 and p50 **(F)**, whole cell lysate protein p105, and **(G)** cytoplasmic NF-κB inhibitory proteins IκBα and IκBβ levels. GAPDH and HDAC1 were used as loading controls for the cytosolic and nuclear extracts, respectively. *P < 0.05 vs. adult (AD) by 1-way ANOVA and Dunnet *post-hoc* test. Values are represented as fold change relative to adult (AD).

The transcription factor NF-κB has been reported to have a central role in the regulation of the innate immune response (43, 44). NF-κB activation induces the expression of pro-inflammatory genes via toll-like receptor (TLR) signaling (28, 45, 46). LPS-mediated activation of the TLR4 induces the degradation of the inhibitory proteins IκBα and IκBβ allowing the nuclear translocation of the NF-κB subunits resulting in the up-regulation of key pro-inflammatory genes (22, 25, 47). We have previously shown that the LPS-mediated hepatic innate immune response in the adult is largely p65 driven compared with the neonate P0 mouse (26). Based on these findings, we decided to explore this relationship at other transitioning time points (late neonate and juvenile) in LPS exposed and unexposed animals. Figure 1 shows the hepatic gene (Figure 1A), whole (Figures 1B,C) and nuclear (Figures 1D,E) protein expression of the NF-κB subunit proteins p65 and p50, hepatic protein expression of NF-κB subunit protein p105 (Figure 1F), and hepatic cytoplasmic NF-κB inhibitory proteins IκBα and IκBβ expression (Figure 1G) in unexposed mice. Hepatic gene expression of *Rela* (p65) and *Nfkb1* (p50) were significantly lower in p0 vs. adult mice (*P* < 0.05). There were not significant differences in other developmental time points compared with adult mice. At the whole protein level, hepatic p65 expression was significantly lower in p0 and p3 vs. adult mice (*P* < 0.05); and at the nuclear level, p65 protein expression was higher in p35 compared with the adult (*P* < 0.05) and expression was undetectable in p0, p3, and p7 mice. There were no differences detected in p50 whole or nuclear protein expression in any developmental group. Likewise, whole hepatic p105 protein and cytoplasmic NF-κB inhibitory proteins IκBα and IκBβ expression were observed at all developmental time points consistently.

### Activation of the Hepatic NF-κB-Mediated Innate Immune Response to IP LPS Is Developmentally Regulated by Subunit p65

**Figure 2:**
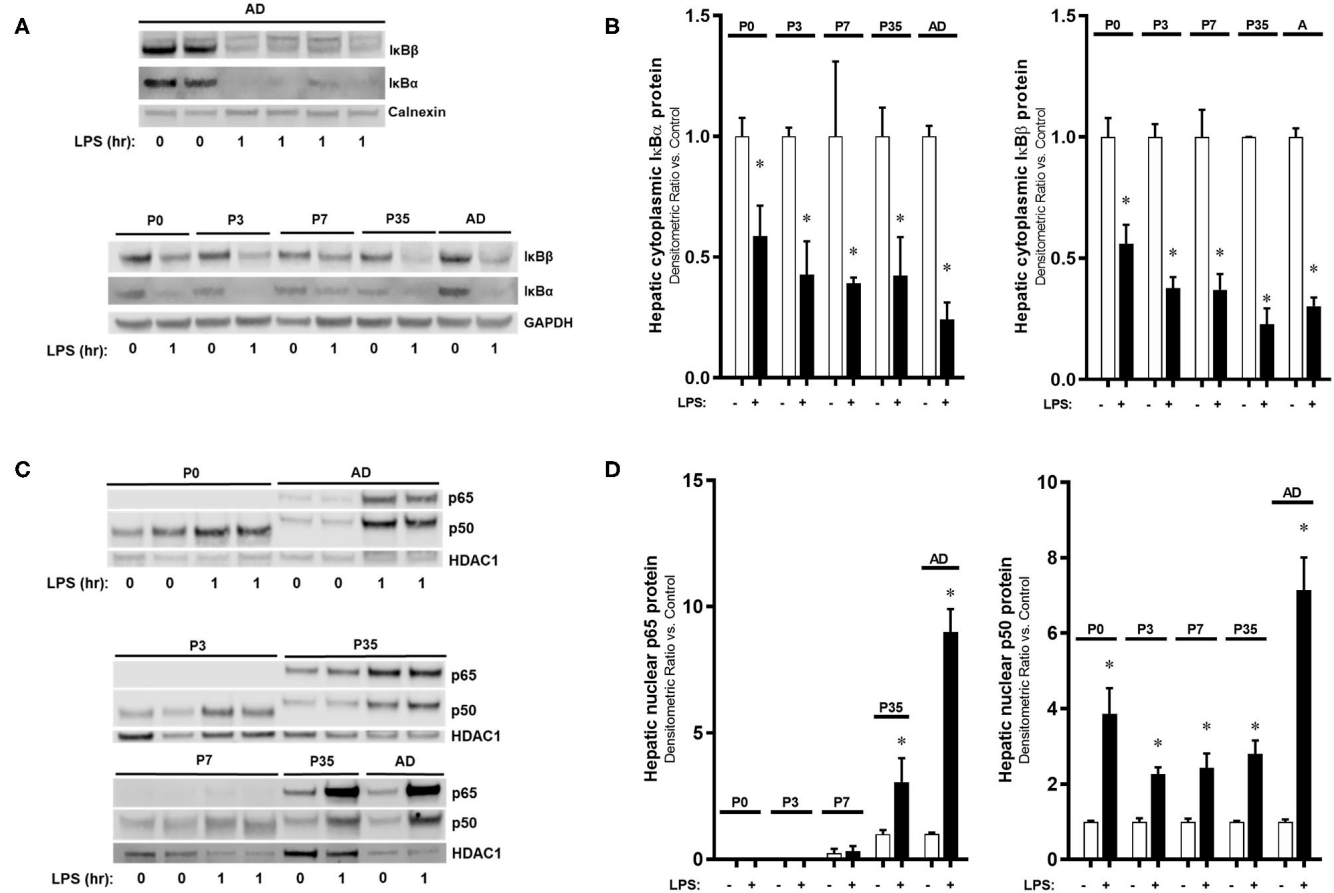
The activation of the hepatic NF-κB-mediated innate immune response to IP LPS is developmentally regulated by the subunit p65. IP LPS-exposed (5 mg/Kg, 1 h) mouse livers (1 h) from different developmental time points were collected and measured for hepatic **(A,B)** cytoplasmic NF-κB inhibitory proteins IκBα and IκBβ, and **(C,D)** nuclear p65 and p50 protein expressions. Loading controls used were calnexin and GAPDH (cytoplasmic), and HDAC1 (nuclear). *P < 0.05 vs. control (0 h) by Student's *T*-test. Values are represented as fold change relative to control.

Having demonstrated significant changes in the expression of *Rela* (p65) and *Nfkb1* (p50) over murine development, we next sought to assess whether there were differences in LPS-induced hepatic NF-κB activation over the same developmental window. We have previously reported that the liver is able to mount an innate immune response to IP LPS as early as 1 h after exposure through NF-κB signaling activation (26) and upregulation of primary response genes (42), which led us to continue studying this initial activation at multiple developmental time points. Exposure to IP LPS resulted in degradation of the cytoplasmic NF-κB inhibitory proteins IκBα and IκBβ occurred within 1 h (Figures 2A,B) with a similar pattern among all developmental ages (*P* < 0.05 vs. control). These data demonstrate that the LPS-induced hepatic NF-κB signaling upstream of IκB inhibitory protein degradation is intact from P0 to adult. Likewise, nuclear translocation of NF-κB p50 subunit 1 h after IP LPS (Figures 2C,D) was significantly present (*P* < 0.05 vs. control) without a significant difference among all ages. However, we found significant differences in LPS-induced p65 nuclear translocation from P0 to adulthood. Specifically, p65 translocation was not detected in p0, p3, and p7 mice when compared with p35 and adult (*P* < 0.05 vs. control). These data demonstrate that despite LPS-induced degradation of the IκB inhibitory proteins at P0 to adulthood, nuclear translocation of key NF-κB subunits was specific to the postnatal age at which exposure occurred.

### IP LPS-Mediated Nuclear Translocation of the NF-κB Subunit p65 Occurs Only in the Adult Mouse Liver

**Figure 3:**
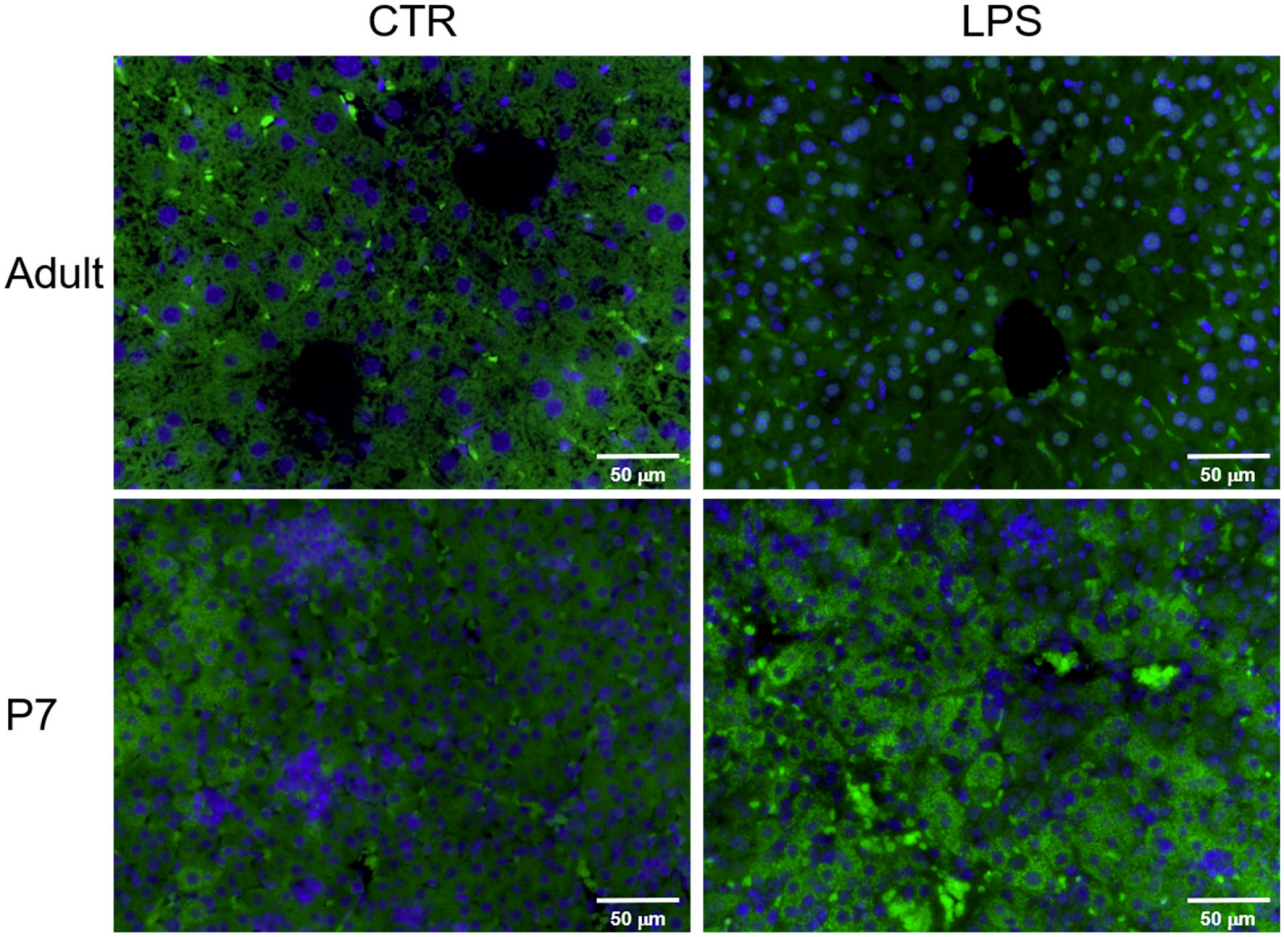
The NF-κB subunit p65 is translocated into the nucleus in the liver of IP LPS-exposed adult mice. Representative histological pictures of control (CTR) and LPS-exposed (5 mg/Kg, 1 h) adult and p7 livers. NFκB subunit p65 was stained in green (FITC) and cell nuclei was stained in blue (DAPI). Internal scale bar to 50 μm. Image at 40x.

Having determined that the NF-κB p65 expression in the LPS-exposed liver is different between the adult and neonate, we finally sought to detect the p65 protein cellular location in the LPS-exposed adult and neonatal liver by immunofluorescence (Figure 3). IP LPS adult liver showed a very distinct p65 nuclear localization (green fluorescence) at 1 h after exposure. In contrast, adult control and p7 mice showed a cytoplasmic p65 localization after LPS exposure. Together, these results demonstrate that upon LPS activation, p65 nuclear translocation only occurs in the adult liver whereas, in the neonate, this expression remains in the cytosol.

### Hepatic DNA-Binding NF-κB Dimers Include p65 in Adults but Not in P7 LPS Exposed Mice

**Figure 4:**
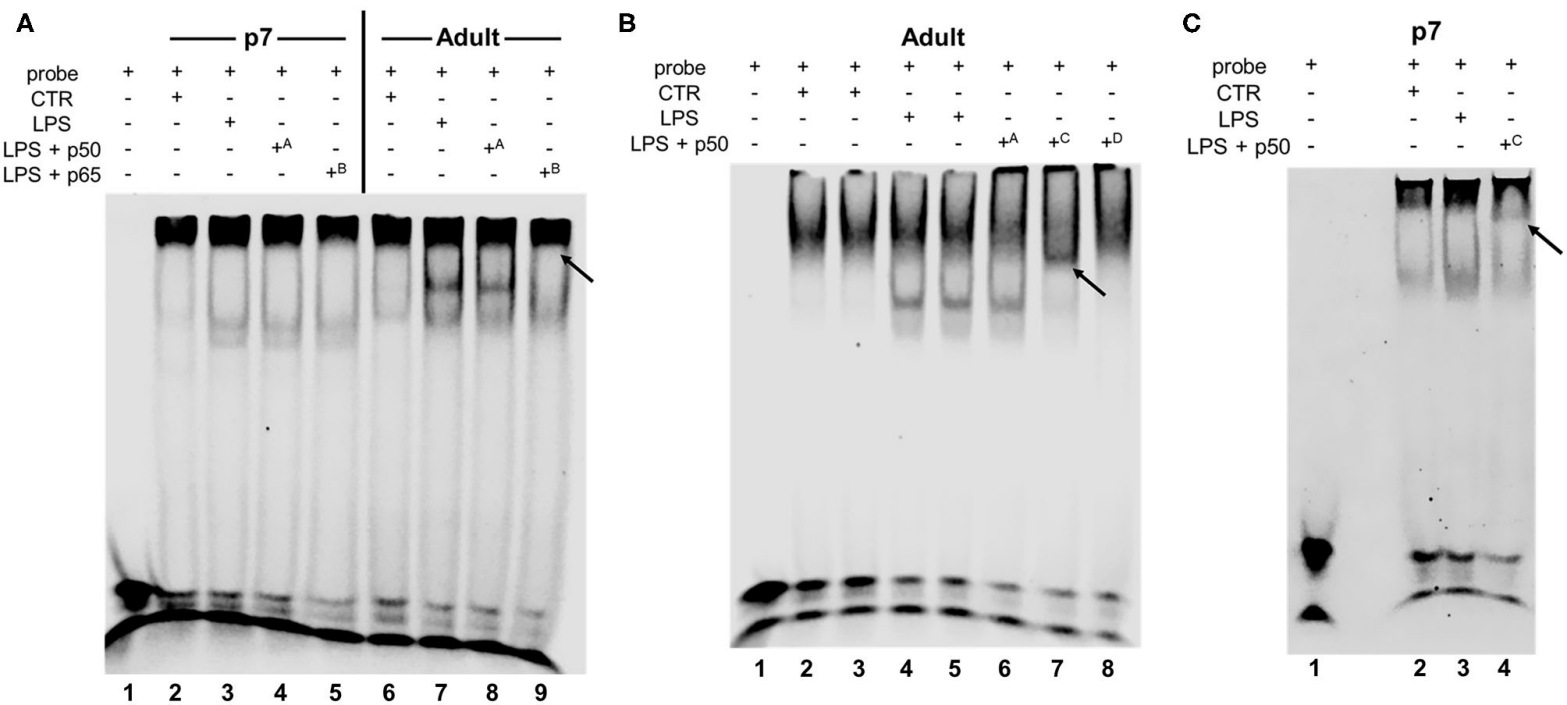
IP LPS induces age-specific hepatic NF-κB activation. Representative EMSA of hepatic nuclear extracts from p7 and adult mice exposed to IP LPS (5 mg/Kg, 1 h) and control (CTR) **(A–C)**. Bands represent NF-κB consensus sequence binding, non-specific binding, free probe, and supershift (black arrows) are labeled. Superscript letters indicate different antibodies used: ^A^ p50 (1.06 mg/mL, ab32360, Abcam, Cambridge, MA, USA), ^B^ p65 (201 μg/mL, #8242, Cell Signaling, Danvers, MA, USA), ^C^ p50 (170 μg/mL, #13586, Cell Signaling, Danvers, MA, USA), and ^D^ p50 (1 mg/mL, #90275, Millipore, Burlington, MA, USA).

Having demonstrated that IP LPS induced differential NF-κB subunit nuclear translocation from P0 to adulthood, we next sought to determine what dimers present in the nucleus were able to bind to DNA. Thus, we performed electrophoretic mobility shift assays (EMSA) on hepatic nuclear extracts obtained from control and LPS exposed (5 mg/kg, 1 h) p7 and adult mice. For this assay, we worked with 3 different p50 subunit antibodies (A, Abcam; C, Cell Signaling; and D, Millipore) and 1 p65 subunit antibody (B, Cell Signaling). Analysis of p7 and adult LPS-exposed livers by EMSA (Figure 4) revealed NF-κB DNA binding at 1 h of exposure compared with their respective controls (Figure 4A lines 3, 7; Figure 4B lines 4, 5; and Figure 4C line 3). Supershift analysis confirmed presence of the NF-κB p50 subunit in both adult and p7 LPS-exposed mice using antibody C (Cell Signaling, Figure 4B line 7 [adult]; Figure 4C line 4 [p7]). In contrast, NF-κB p65 subunit supershift was only detected in LPS-exposed adult compared with p7 mice (Figure 4A line 9 [adult]; Figure 4A line 5 [p7]). These results confirm that LPS induces nuclear translocation of NF-κB dimers that contain p50 but not p65 at p7, while NF-κB dimers contain both p50 and p65 in adults.

### Endotoxemia Induces Developmentally-Regulated Hepatic Expression of Primary Pro-Inflammatory Genes

**Figure 5:**
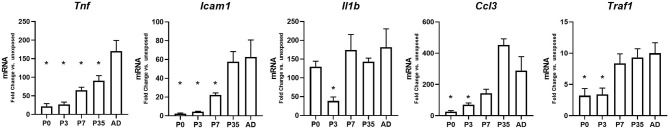
Endotoxemia induces developmentally-regulated hepatic expression of primary pro-inflammatory genes. Hepatic gene expression levels for primary innate immune pro-inflammatory genes 1-h post IP LPS at different developmental time points. *P < 0.05 vs. adult (AD) by 1-way ANOVA and Dunnet *post-hoc* test. Values are represented as fold change compared to their own unexposed control.

Exposure to LPS induces a NF-κB-mediated pro-inflammatory signaling in the liver, and we have previously described that this response is attenuated in neonates compared with adults (26). However, how this response matures over time has never been described. Having observed that IP LPS induces a p65-dependent hepatic innate immune over murine maturation, we then sought to determine whether this change was associated with increasing hepatic expression of p65 dependent pro-inflammatory genes. Thus, we interrogated the expression of key primary response, pro-inflammatory genes (*Tnf, Icam1, Il1b, Ccl3*, and *Traf1*) whose promoter regions are known to interact with both NF-κB subunits p65 and p50 (33), the NF-κB subunits available to bind DNA in LPS-exposed adult mice as demonstrated by EMSA in Figure 4. Figure 5 shows comparison in gene expression levels between LPS-exposed vs. unexposed to determine the different NF-κB activation levels of stimulation across development. We found that compared to LPS-exposed adults, LPS-induced gene expression was significantly lower at p0, p3, p7, and p35 (*Tnf);* p0, p3, and p7 (*Icam1*); p3 (*Il1b*); and p0 and p3 (*Ccl3 and Traf1*).

### The NF-κB Subunit p65 Binds the *Icam1* Promoter in the LPS-Exposed Adult Liver

**Figure 6:**
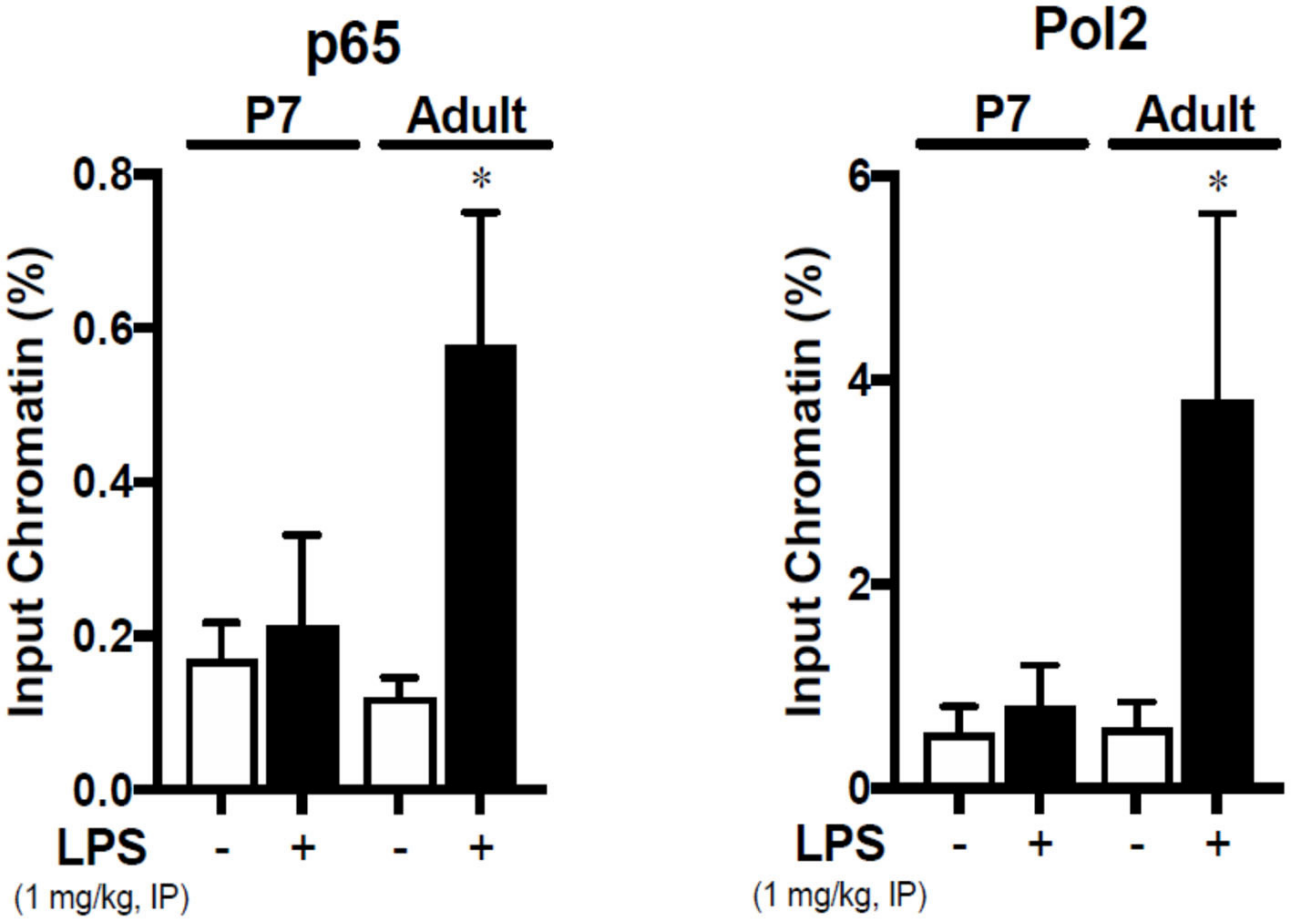
The NF-κB subunit p65 is bound to the *Icam1* promoter in the liver of IP LPS-exposed adult mice. Adult and p7 mice were exposed to IP LPS (5 mg/Kg, 1 h), and whole liver homogenates were subjected to chromatin immunoprecipitation with anti-p65 (SC-372, Santa Cruz Biotechnology, Dallas, TX, USA) and analyzed by RT-qPCR targeting the *Icam1* promoter. RNA Polymerase 2 (Pol2) was used to validate the assay. *P < 0.05 vs. control (0 h) by Student's *T*-test. Values are represented as input chromatin percentage of RT-qPCR for the amplification of the *Icam1* promoter run in triplicate.

Having observed the effects of IP LPS on the hepatic expression of primary pro-inflammatory genes and NF-κB p65 activation, we next evaluated whether there is a direct relationship between LPS-induced p65 nuclear translocation and the expression of *Icam1* in the adult liver. *Icam1* is an important pro-inflammatory mediator involved in the recruitment of leukocytes into different tissues (48–50) and is present in the liver in the sinusoidal lining cells (51, 52), hepatocytes (53, 54), and Kupffer cells (54). Likewise, *Icam1* gene promoter has been shown to strongly interact to the NF-κB subunit p65 in LPS-exposed human monocytes (33), macrophages (55), and alveolar cells (56, 57) *in-vitro*. We found that 1 h following IP LPS exposure, NF-κB p65 subunit bound the *Icam1* promoter in the adult liver (*P* < 0.05 vs. control), and could find no evidence of p65 binding to the *Icam1* promoter in LPS-exposed p7 mice (Figure 6). RNA Polymerase II interaction with *Icam1* promoter followed the same pattern thus validating our results. Our data demonstrate that expression of LPS-induced hepatic *Icam1 gene* expression is developmentally regulated by NF-κB subunit p65.

## Discussion

The main goal of this study was to assess the LPS-induced NF-κB-mediated innate immune activation in the mouse liver across development. This study reveals that the maturation of the NF-κB-mediated hepatic innate immune system is dependent of the actions of *Rela* (p65) in mice. The present work shows three important findings: (1) LPS-induced hepatic NF-κB subunits p65 and p50 activation does only occur in the juvenile (p35) and adult mouse; (2) neonatal hepatic NF-κB innate immune response to IP LPS does not involve p65, but does depend on the NF-κB subunit p50; and (3) the NF-κB subunit p65 activation is transcriptionally active and can be found bound to the promoter region of pro-inflammatory genes that are expressed to a greater extent in in the LPS-exposed juvenile and adult compared to neonate mouse.

After stimulation of the innate immune system, the NF-κB response is highly dependent on the levels and stability of the subunit p65 (58). This subunit has the primary role of inducing pro-inflammatory gene responses (59) and also amplifies the pro-inflammatory signaling through an autoregulatory loop mechanism (60). In this work, we were able to detect hepatic *Rela* (p65) and *Nfkb1* (p50) gene and protein expression levels in the unexposed mouse across all developmental ages. The presence of hepatic NF-κB p50 protein was supported by p105 protein detection in liver lysates at all developmental ages consistently. However, after LPS stimulation, the NF-κB subunit p65 faces a different extent of activation and activity that is dynamic as the mouse matures. Here, we present data demonstrating that exposure to IP LPS induced nuclear translocation of both p50 and p65 in the livers of juvenile and adult mice, but only p50 translocation was detected in the neonate. We have previously reported that the neonatal mice (p0) do not show hepatic p65-NF-κB signaling compared with the adult at 1 h post IP LPS exposure (26). The present work builds on our previous findings by demonstrating that LPS-induced NF-κB subunit p65 nuclear translocation, DNA binding and transcriptional activity does not occur at developmental ages p3 and p7 after LPS exposure. All these findings were validated by Western blot, immunostaining against p65 subunit in the LPS-exposed adult vs. neonate (p7) mouse liver, EMSA and ChIP. Our data show that NF-κB subunit p65 hepatic nuclear translocation only occurs in the adult after LPS exposure. In contrast, p7 neonatal mice showed hepatic cytosolic subunit p65 expression but there was no nuclear translocation after exposure.

This study shows that the LPS-induced hepatic expression of NF-κB-associated pro-inflammatory genes (*Tnf, Icam1, Ccl3*, and *Traf)* is greater in the adult compared with the neonate mouse. Previous studies have shown that cytokines such as TNF-α, CCL3, and IL1β along with others have been shown to be significantly reduced in neonatal monocytes (61) and systemically (11, 62) when exposed to LPS. There are few reports that describe whether this situation also occurs similarly in the liver. For instance, Le Rouzic et al. found that the hepatic innate immunity is suppressed in the p1 neonatal mice compared with juveniles (p21) and adults (p70) by measuring basal *TLR4, CD14*, and *MD2* gene expression in rats (63). Furthermore, Nakagaki et al. reported a significant higher *Tlr4, Itgam* (CD11b, macrophage marker), and a reduced *Nr1h3* (NR1, regulator of macrophage function) hepatic gene expression in the neonatal mice when exposed to LPS (64) indicating an insufficient innate immunity maturation. Our findings are consistent with these reports, and also reveals that the LPS-induced hepatic downstream pro-inflammatory cascade remains attenuated at p3 and p7 ages. Likewise, our data show that increases in LPS-induced hepatic pro-inflammatory cytokine upregulation that occur from birth to adulthood are associated with significant differences in the nuclear translocation and DNA binding of the NF-κB subunit p65. We have shown that nuclear p65 binds to DNA sequences in the promoter region of the *Icam1* gene after LPS exposure as shown by ChIP data. This result fits well with previous reports describing a large distribution of p65 DNA binding sites in different cells upon TLR4 activation that leads to up-regulation of pro-inflammatory responses (33, 65, 66). Together, our findings indicate that the neonatal mouse does not have a fully matured hepatic innate immunity due to a reduced NF-κB subunit p65 pro-inflammatory activity.

The mechanisms how the NF-κB p65 subunit induces an innate immune pro-inflammatory response are well-described. This subunit is ubiquitous (22), considered the main target for phosphorylation by numerous kinases (59), and transcriptionally active in most tissues as homodimers (p65/p65) or heterodimers with NF-κB subunits p50 and c-rel (22). Also, the NF-κB subunit p65 profound pro-inflammatory actions are strongly involved in organ defense against damage-associated (DAMPs) and pathogen-associated (PAMPs) molecular patterns (22, 67). This molecule is very stable (68, 69) and has regulatory effects on the subunit p50 (70) and NF-κB inhibitory proteins activity (60). Likewise, NF-κB subunit p65 can be post-translational modified through phosphorylation (71), acetylation (72), isomeration (73), methylation (74), and ubiquitination (75), and exert other cellular functions which adds more complexity to its role. However, the exact mechanisms by which p65 activity is reduced in the neonatal liver are unknown. We can speculate that, due to its unique and essential role in clearing antigens systemically, hepatic p65 is associated with strong pro-inflammatory responses that cannot be regulated by the neonatal immune system, yet. Without correct regulatory mechanisms, NF-κB p65-dependent activation of the hepatic innate immune system may lead to severe organ damage in the neonate.

In contrast to some reports (76, 77), we found that neonates are able to mount an innate immune response to IP LPS but in a lesser extent compared with the adult mouse, possibly due to an exclusive subunit NF-κB p50-dependent mechanism. Contrary to p65 actions, NF-κB p50 subunit is reported to have transcriptional repressor effects on the innate immune signaling (58). However, NF-κB subunit p50 can become transcriptionally active through an association with other NF-κB inhibitory proteins such as a B-cell lymphoma 3-encoded protein (Bcl3) (78–80), and IκBζ (81– 83) and induce immunoregulatory actions. Whether these mechanisms are active in the maturing liver is an area of active study. Additionally, we found in this study that hepatic NF-κB subunit p50 is present in all developmental ages and can be largely translocated after LPS exposure. There are no reports that describe how the NF-κB subunit p50 develops or matures over time in the liver, but along with its immunosuppressor effects (58, 84), subunit p50 has been reported to have a relevant role in other cellular processes, such as organogenesis (85), neuroplasticity (86), anti-aging processes (87), hepatic tumor suppressing (88), and liver metabolism (89, 90). We believe that NF-κB subunit p50 role is essential in the liver since its expression is pretty consistent among all ages in mice. Studies with NF-κB subunit p50 K.O. animal and short hairpin RNA on specific cell lines for gene knockdown can provide relevant information to confirm the functional role of subunit p50 during development and its activation when exposed to different stressors.

Our studies are limited in that we did not identify the specific liver cell populations involved in LPS-mediated NFκB p65 activation and assess their contribution to the hepatic innate immune response through *in-vitro* studies. Of note, reports describe that the neonatal liver is myeloid-enriched in granulocytes, monocytes, and immune cell precursors, all of them with a reduced Th1 activity (64). It is possible that the difference in hepatic cell populations between the neonatal and adult mouse might affect the p65-mediated NFκB signaling after LPS exposure. Furthermore, the aim of this study was to assess the NF-κB-mediated hepatic innate immune response to LPS at 1 h (acute) and did not assess any later pro-inflammatory or systemic signaling effects. However, for the first time, this work identified notable differences in the initial activation of the NF-κB signaling in the liver across development and will provide relevant data that will be used for testing the response to LPS at different range of exposures (doses) and later time points. Likewise, this work showed that NF-κB subunit p65 nuclear activation is transcriptionally active in the juvenile and adult mice, but this subunit might not be the only one that can induce NF-κB-mediated innate immune activation in the liver. There is also the need to assess the contribution of the NF-κB subunit c-Rel, which is present in the liver (91) and is reported to have immunosuppressive actions (58, 91), as well as the non-canonical NF-κB pathway activation through the nuclear translocation of NF-κB subunits p52 and RelB (92). We only detected a small increment in hepatic nuclear p52 expression in the adult but did not observe any nuclear translocation in the neonate (p7) after LPS exposure (Supplemental Figure 2). Furthermore, the degree of response to an inflammatory stimulus can be sex-specific (93, 94). The present work only used males, but future studies should focus on describing the female NF-κB-mediated innate immune response to LPS as well as the factors that can influence this signaling (e.g., estrous cycle) at different developmental time points.

We conclude that the maturation of the NF-κB transcriptome is regulated by the subunit p65 in the liver. We have shown that an inflammatory challenge (IP LPS) induces a robust upregulation of hepatic NF-κB target genes identified to be subunit p65 dependent in the adult. Nuclear extracts from LPS-exposed adult livers showed that NF-κB subunit p65 becomes transcriptionally active and is able to bind the promoter region of pro-inflammatory genes in the juvenile (p35) and adult mouse thus promoting gene transcription. In contrast, LPS-exposed neonates lack hepatic NF-κB subunit p65 activation and this is associated with attenuated expression of key pro-inflammatory NF-κB regulated genes. The role of hepatic NF-κB subunit p65 on the development of the hepatic innate immunity might provide novel insights in how to treat neonatal sepsis by creating therapeutic approaches targeting specific NF-κB subunits.

## Data Availability Statement

All datasets presented in this study are included in the article/Supplementary Material.

## Ethics Statement

The animal study was reviewed and approved by The University of Colorado Institutional Animal Care and Use Committee (00457).

## Author Contributions

MZ and CW: conception and design of research, data analysis and interpretation, and Elaboration of manuscript and figures. MZ, CW, LN, RD, and LZ: data collection. All authors have reviewed the manuscript and agreed with its submission.

## Conflict of Interest

The authors declare that the research was conducted in the absence of any commercial or financial relationships that could be construed as a potential conflict of interest.
