# Supplementary figures and images for: Maturation of the Acute Hepatic TLR4/NF-κB Mediated Innate Immune Response Is p65 Dependent in Mice
Source: Front Immunol. 2020 Aug 21;11:1892. doi: 10.3389/fimmu.2020.01892 (PMC7472845; doi:10.3389/fimmu.2020.01892)

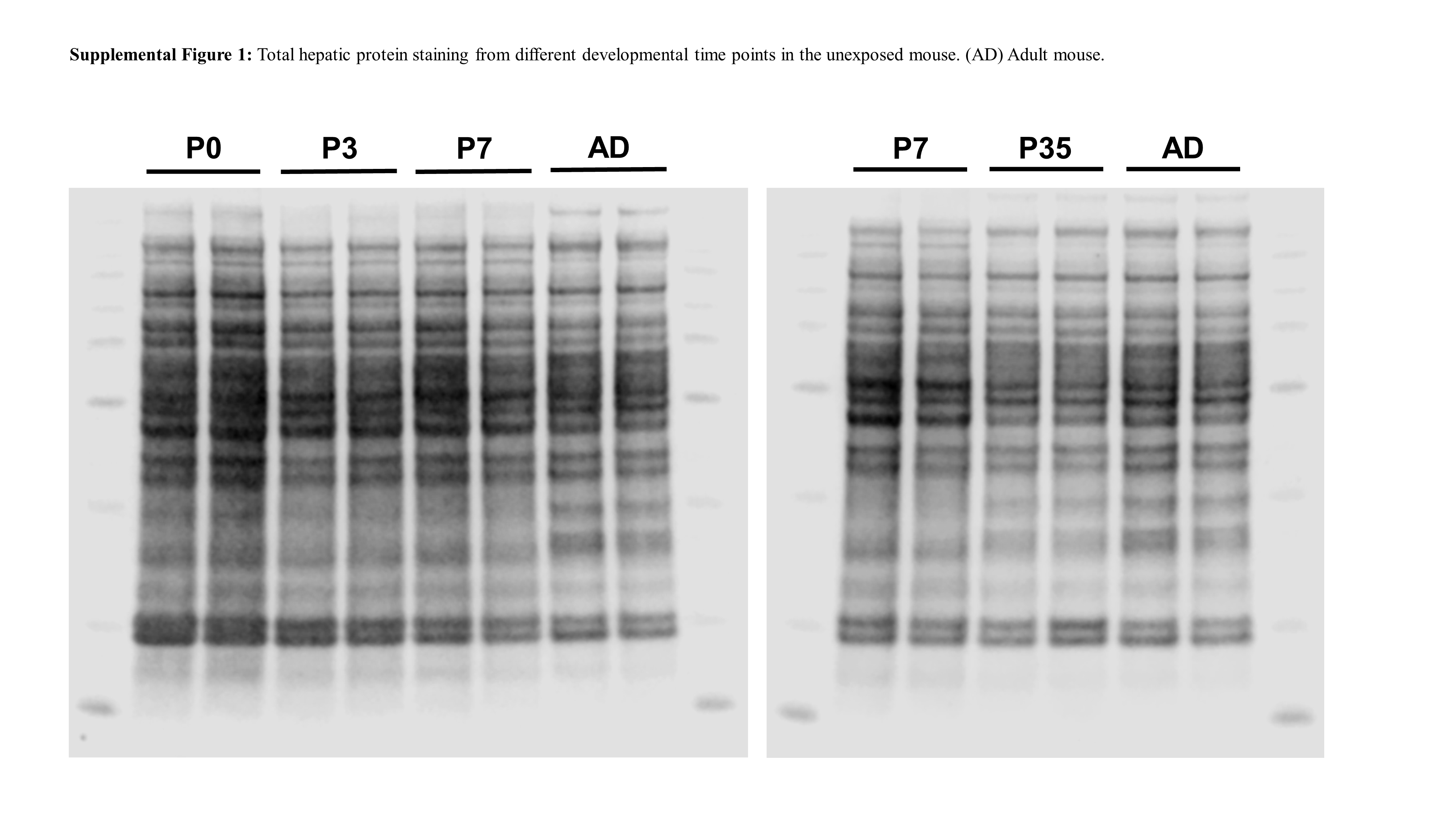

Supplement: Supplementary file 1 [file Image_1.TIF]

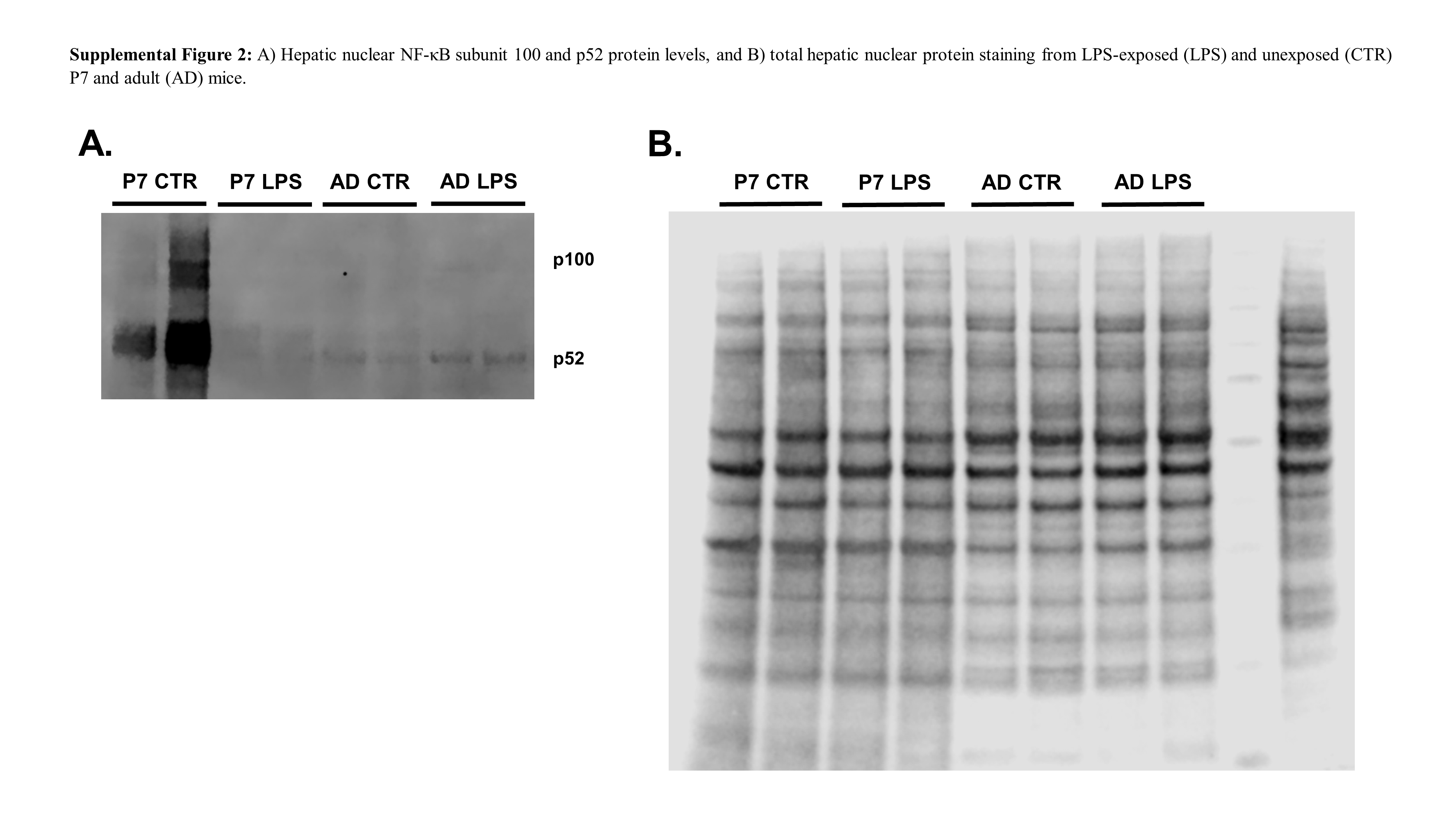

Supplement: Supplementary file 2 [file Image_2.TIF]
